# The RNA structure of the major splice donor site controls HIV-1 splicing

**DOI:** 10.1186/1742-4690-10-S1-P62

**Published:** 2013-09-19

**Authors:** Nancy Mueller, Ben Berkhout, Atze Das

**Affiliations:** 1 Laboratory of Experimental Virology, University of Amsterdam, Amsterdam, The Netherlands

## Background

The unspliced HIV-1 RNA is used as mRNA for translation of the Gag and Pol proteins and packaged as genomic RNA in the virion. In addition, HIV-1 produces more than 40 differentially spliced transcripts that encode the other viral proteins. Regulation of splicing is essential for the production of all unspliced and spliced viral RNAs. The 5’ leader region of the HIV-1 RNA contains the major 5’ splice site (5’ss) that is used in the production of all spliced RNAs. This splice donor (SD) region can fold a stem-loop structure and the stability of this hairpin may influence splicing by restricting the accessibility of the 5’ss for the splicing machinery. To test this hypothesis, the thermodynamic stability of the SD hairpin was varied through mutation and the effect on RNA splicing was analyzed.

## Materials and methods

The SD hairpin in the HIV-1 leader RNA region was stabilized or destabilized through mutation in the context of the HIV-1 molecular clone LAI. The effect on virus replication was measured upon infection of the SupT1 T cell line. The mutations were also introduced into an LTR-luciferase plasmid. Upon transfection of these reporter constructs into 293T cells, the efficiency of splicing at the major 5’ss was analyzed by luciferase activity assays and RNA analysis.

## Results

Both stabilization and destabilization of the SD hairpin reduced HIV-1 replication. The LTR-luciferase experiments demonstrated that stabilization of the SD hairpin reduced 5’ss usage, whereas destabilization increased splicing at this position.

## Conclusions

The stability of the SD hairpin controls the level of splicing at the major 5’ss. The wild-type SD hairpin structure inhibits 5’ss usage to a level that results in an optimal balance between unspliced and spliced RNAs for efficient virus replication.

